# Cultural and Sex-Related Differences in Free-Word Associations with “Sweets”: A Multinational Online Study

**DOI:** 10.3390/nu18111771

**Published:** 2026-05-30

**Authors:** Nicole Avena, Réka Erika Kovács, Angéla Somogyi, Ricardo de la Vega, Attila Szabo

**Affiliations:** 1 Department of Neuroscience, Icahn School of Medicine at Mount Sinai, New York, NY 10029, USA; nicole.avena@mssm.edu; 2 Health Psychology, Princeton University, Princeton, NJ 08544, USA; 3 Faculty of Health and Sport Sciences, Széchenyi István University, 9026 Győr, Hungary; kovacs.reka.erika@sze.hu (R.E.K.); somogyi.angela@sze.hu (A.S.); 4 Departamento de Educación Física, Deporte y Motricidad Humana, Universidad Autónoma de Madrid, 28049 Madrid, Spain; ricardo.delavega@uam.es

**Keywords:** BMI, cross-cultural, eating behavior, eating disorders, free-word association, sex differences

## Abstract

**Background**: Free-word association (FWA) captures the most accessible lexical responses to a stimulus, providing a window into automatic cognitive representations of food categories that may differ across cultures and between sexes. **Objectives**: To examine whether the dominant cognitive associations evoked by the word “sweets” differ across three language groups (Hungarian, English, Spanish), whether they vary by sex, and whether they relate to body mass index (BMI) and self-reported eating disorder risk. **Methods**: A total of 1349 participants completed an online survey including a single FWA prompt. Responses were classified into 10 semantic categories and analyzed using chi-square tests. Effect sizes (Cramér’s V) were reported for all tests. Height and weight were converted to uniform metric units, and BMI was calculated. **Results**: The association profile differed significantly across language groups (χ^2^[18] = 210.05, *p* < 0.001, V = 0.28). Chocolate dominated Hungarian responses, while Baked goods/Desserts and Sugar/Candy dominated English, and Positive emotion and Baked goods/Desserts predominated among Spanish speakers. Sex differences were significant overall (χ^2^[9] = 43.72, *p* < 0.001, V = 0.18). BMI distributions differed markedly across nations (χ^2^[6] = 157.17, *p* < 0.001, V = 0.26), and sweets categories were significantly associated with eating disorder risk (χ^2^[27] = 48.04, *p* = 0.008, V = 0.11); however, this result should be interpreted with caution given the extreme skew toward the lowest-risk category [84.2%], with Negative/Health associations overrepresented among higher-risk participants. **Conclusions**: Cultural context plays a significant role in shaping automatic cognitive associations with sweet foods. The exploratory association between sweets categories and self-reported eating disorder risk warrants further investigation using validated instruments before any substantive conclusions can be drawn.

## 1. Introduction

Sweet foods are among the most widely consumed and behaviorally significant food categories globally, with their overconsumption linked to obesity, type 2 diabetes, and dysregulated eating patterns [1,2,3]. Understanding the automatic cognitive associations that individuals form with sweet foods is of considerable theoretical and clinical relevance, as such representations underlie cue-driven eating and food cravings [4,5]. Sweet food cues can trigger appetitive responding even in the absence of hunger [6], suggesting that associative learning shapes consummatory behavior through pathways that operate independently of homeostatic need [7]. Tiffany’s (1990) cognitive model of drug urges provides a useful theoretical framework for understanding food-related associations. According to this model, repeated exposure to appetitive substances activates automatic cognitive schemata—well-rehearsed action plans stored in memory—that can be triggered by cues without conscious intention [4]. Extending this framework to food cognition, automatic associations with food categories such as “sweets” may reflect overlearned semantic representations that are activated rapidly upon exposure to category cues. Unlike validated implicit measures (e.g., the Implicit Association Test), free-word association captures the most accessible lexical response to a stimulus, which may reflect lexical frequency and cultural salience as well as underlying cognitive organization.

Free-word association (FWA) is a low-burden method for probing spontaneous, pre-reflective cognitive responses about food [8,9]. It captures the single most salient lexical association, providing a complementary window into which food concepts are most cognitively accessible in response to a category cue. In research on eating behavior, FWA has been used to characterize lay food categories, trace hedonic versus health-oriented food representations, and identify culturally embedded product associations [10,11].

Food associations are substantially shaped by cultural, linguistic, and environmental context [12,13]. A concept as ubiquitous as “sweets” may carry strikingly different connotations across linguistic communities—evoking a specific product in one context (e.g., chocolate) and a broader evaluative response in another (e.g., “delicious” or “sugary”). Thus, cross-language comparisons of single-word responses must be interpreted cautiously, as observed differences may reflect lexical accessibility, word frequency, and translation asymmetries rather than deeper cognitive structures. Furthermore, the Spanish word ‘dulce’ exhibits well-documented polysemy that extends to interpersonal kindness, potentially introducing systematic variation in how Spanish-speaking participants interpret the FWA prompt.

Sex differences in food cognition are well established. Women report stronger food cravings, especially for sweet and high-carbohydrate foods, and place greater hedonic value on food experiences than men [14,15]. Chocolate cravings are more common and intense among women than men in Western samples [16,17,18]. Cross-cultural evidence from Rodríguez et al. (2007) [19] further demonstrates that chocolate craving patterns vary between English- and Spanish-speaking women, highlighting the interplay between sex and culture in food-specific craving. However, it remains unclear whether these differences appear in the automatic lexical associations that women and men form with broad food categories, and whether sex-related patterns are consistent across cultures.

Beyond culture and sex, individual differences in body weight status and eating pathology may also shape how people conceptualize sweet foods [20,21]. Individuals with higher BMI or elevated eating disorder risk may associate sweets with health concerns, craving, or loss of control rather than with pleasure or specific food products [22]. However, the relationship between automatic food associations and these individual-difference variables has received limited attention in the cross-cultural FWA literature.

The current study addressed three research questions using data from a multinational online survey (*N* = 1349) across three language communities. RQ1: Do cognitive associations with “sweets” differ significantly across Hungarian, English, and Spanish speakers? RQ2: Are response category profiles significantly different between women and men? RQ3: Are sweet-food association categories related to BMI category and self-reported eating disorder risk?

## 2. Methods

### 2.1. Participants and Procedure

The study received ethical approval (Permission No. SZE/ETT-39/2025 [vii. 8.]) from the Scientific Ethics Board of Széchenyi István University. Participants were recruited via social media (Facebook, Instagram, Threads, LinkedIn) in Hungary, the United States (NY, NJ), and Spain. This convenience sampling strategy yields a self-selected, non-representative sample with unknown demographic biases, limiting generalizability. A total of *N* = 1349 participants completed the survey (English: *n* = 544, 40.4%; Hungarian: *n* = 461, 34.2%; Spanish: *n* = 344, 25.5%). Women comprised 68.0% of the sample (*n* = 917), men 31.7% (*n* = 428), and four participants (0.3%) reported other sex. The sex imbalance was particularly pronounced in the English subsample (women: *n* = 458, 84.2%; men: *n* = 83, 15.3%), which limits sex-stratified analyses for that group. The research was conducted in accordance with the Declaration of Helsinki; participants provided informed electronic consent prior to participation.

### 2.2. Free-Word Association Task

The FWA task presented participants with the prompt: “What comes to mind when you see or hear the word ‘sweets’?” Participants typed the first word or phrase that came to mind, with no time limit. The task was presented in the participant’s survey language (Hungarian, English, or Spanish). It should be noted that the absence of a time limit and the allowance of multi-word responses reduce standardization and may affect comparability across respondents. Furthermore, the stimulus term is not semantically equivalent across the three languages: Hungarian “édesség” is a broad confectionery term, English “sweets” carries both food-category and evaluative connotations, and Spanish “dulce” exhibits polysemy extending to interpersonal qualities.

### 2.3. Response Coding

FWA responses were categorized into 10 mutually exclusive semantic categories through a combination of AI-assisted classification (Claude AI, Opus v. 4.6 Extended) and researcher verification. Categories were developed inductively by two researchers who independently reviewed the full response corpus and proposed initial groupings through iterative discussion. The resulting 10 categories represent three broad semantic domains: food-specific referents (Baked goods/Desserts, Chocolate, Food [general], Mixed food items, Sugar/Candy), evaluative/health-related responses (Negative/Health, Other), and affective/motivational responses (Craving/Desire, Positive emotion, Reward/Comfort). AI classification was used as a first-pass sorting tool; all AI-assigned categories were independently verified by two researchers, and disagreements were resolved through consensus discussion. Inter-rater agreement was assessed on a 20% random subsample, yielding Cohen’s κ = 0.87, indicating strong agreement. Multi-word responses were categorized by their dominant referent. Responses listing multiple specific food types (e.g., “chocolate, cookies, and candy”) were classified as Mixed food items.

### 2.4. Anthropometric Measures and BMI

Participants self-reported their height and weight. Self-reported anthropometric data are known to introduce systematic bias, with height typically overestimated and weight underestimated, potentially attenuating BMI-related associations. Because the survey was administered in three languages, measurement units varied: English-language respondents typically reported in imperial units (inches, pounds), while Hungarian and Spanish respondents used metric units (centimeters, kilograms). All values were converted to meters and kilograms, respectively. When metric markers (cm, kg) were present in English-language responses, values were treated as metric. BMI was calculated as weight (kg)/height (m^2^). Cases with implausible BMI values (<12 or >60) were excluded (*n* = 150). BMI was categorized as: Underweight (<18.5), Healthy (18.5–24.9), Overweight (25.0–29.9), and Obese (≥30.0).

### 2.5. Self-Reported Eating Disorder Risk

Eating disorder risk was assessed with a single screening item scored on a 4-point scale. The question was: Are you currently struggling with an eating disorder? The answer options were: (1) yes, and I am receiving therapy; (2) yes, but I am not receiving therapy; (3) no, but I should seek help (it is possible that my symptoms indicate an eating disorder); (4) no. This single-item measure has not been psychometrically validated and conflates diagnosis, treatment status, and subjective concern into a single response. It is susceptible to subjective interpretation and social desirability bias. Results based on this measure should be considered exploratory; future studies should employ validated multi-item instruments (e.g., EAT-26, SCOFF). Household size was also recorded on a 4-point scale (The question was: How many people live in your household? Answer options were: (1) I live alone; (2) 2; (3) 3–4; (4) 5 or more).

### 2.6. Statistical Analysis

Chi-square tests of independence were used for all categorical analyses. Cramér’s V was calculated as an effect size measure for all chi-square tests, with values of approximately 0.10, 0.30, and 0.50 interpreted as small, medium, and large effects, respectively. For RQ1, a 10 × 3 contingency table (semantic category × language group) was analyzed. For RQ2, a 10 × 2 table (category × sex) was tested for the full sample and for women/men only. For RQ3, category × BMI category and category × eating disorder status tables were analyzed for the full sample and for each nation separately. Household size was also examined. No correction for multiple testing was applied, and no multivariate modeling was conducted. These analytic choices limit the ability to distinguish independent effects of language, sex, and BMI from confounded associations. The extreme skewness in the eating disorder variable (84.2% in the lowest-risk category) and the resulting small cell counts in higher-risk categories compromise the chi-square assumptions and preclude reliable multivariate modeling for that variable. All analyses were performed using Python 3.11 (SciPy 1.11). Statistical significance was set at α = 0.05.

## 3. Results

The final sample comprised 1349 participants (68.0% women; mean age was not collected). BMI was calculable for 1199 participants (88.9%). The distribution of eating disorder risk was heavily skewed: 84.2% (*n* = 1136) reported no eating disorder concerns (ED = 4), while only 1.5% (*n* = 20) reported the highest risk level (ED = 1).

### 3.1. Language Group Differences in Sweets Associations (RQ1)

**Figure 1:**
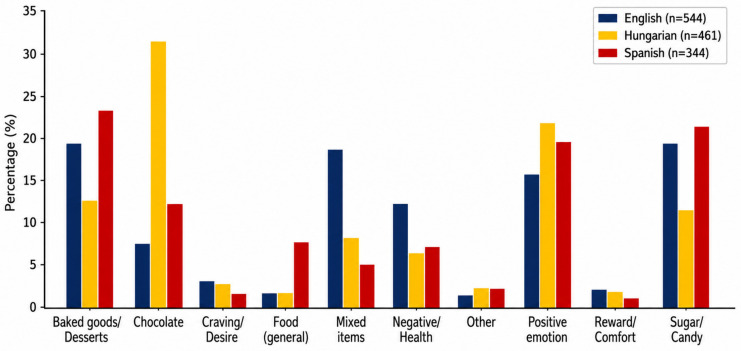
Distribution of sweets association categories by language group. Bars represent the percentage of responses within each language group assigned to each category. χ^2^(18) = 210.05, *p* < 0.001.

The chi-square test confirmed that semantic association profiles differed significantly across language groups (χ^2^[18, *N* = 1349] = 210.05, *p* < 0.001, Cramér’s V = 0.28), with a medium effect size. This effect likely reflects both underlying cultural differences and language-specific lexical accessibility patterns. The frequencies are presented in Table 1. Chocolate was markedly overrepresented among Hungarian speakers: 31.5% of all Hungarian responses named chocolate, compared with 7.3% of English and 12.2% of Spanish responses. Positive emotion was the most frequent category overall (18.7%), with relatively even distribution across nations. English speakers produced the highest proportions of Baked goods/Desserts (19.3%), Mixed food items (18.6%), Sugar/Candy (19.3%), and Negative/Health (12.1%). Spanish respondents showed the highest frequency in Food (general) (7.6%). The Negative/Health category was notably more prevalent among English speakers (12.1%) than among Hungarian (6.3%) or Spanish (7.0%) speakers. Distributions are presented in Figure 1.

### 3.2. Sex Differences in Sweets Associations (RQ2)

**Figure 2:**
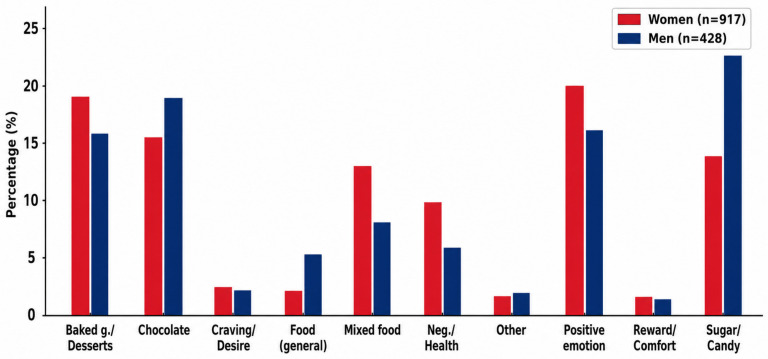
Distribution of sweets association categories by sex (full sample). Women (*n* = 917) showed higher proportions in Baked goods (Baked g.)/Desserts, Mixed food items, and Negative/Health; men (*n* = 428) in Sugar/Candy and Chocolate. χ^2^(9) = 43.72, *p* < 0.001.

The association between meaning categories and sex was significant (χ^2^[9, *N* = 1345] = 43.72, *p* < 0.001, Cramér’s V = 0.18, indicating a small-to-medium effect), excluding four participants who reported other sex. Frequencies by sex within each nation are presented in Table 2. Women showed higher proportions of Baked goods/Desserts (19.2% vs. 15.7%), Mixed foods (13.2% vs. 8.2%), and Negative/Health (10.0% vs. 6.1%) responses, while men showed higher proportions in Sugar/Candy (24.1% vs. 13.8%) and Chocolate (18.9% vs. 15.7%) (Figure 2). The sex imbalance within the English sample (women: *n* = 458; men: *n* = 83) limits within-nation interpretation for that group.

### 3.3. BMI Distributions by Nation

BMI was calculable for 1199 participants. The distribution of BMI categories differed significantly across nations (χ^2^[6] = 157.17, *p* < 0.001; Cramér’s V = 0.26, Table 3). The English-speaking sample had a markedly higher proportion classified as Obese (30.3%) than the Hungarian (8.9%) and Spanish (6.8%) samples. The Hungarian sample had the highest proportion in the Healthy category (68.6%), followed by the Spanish sample (71.5%) and the English sample (40.8%). Mean BMI was 27.96 (SD = 7.51) for English speakers, 23.34 (SD = 4.57) for Hungarian speakers, and 23.69 (SD = 3.96) for Spanish speakers.

### 3.4. Sweets Associations and Eating Disorder Risk (RQ3)

**Figure 3:**
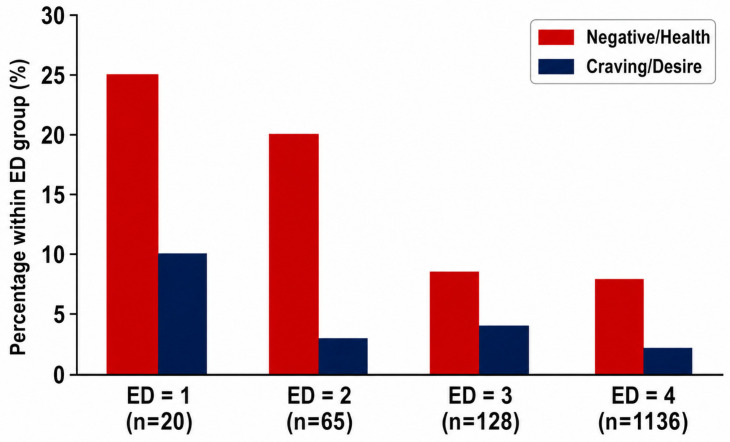
Percentage of Negative/Health and Craving/Desire responses by eating disorder risk level. Participants at higher risk (ED = 1, 2) were substantially overrepresented in Negative/Health associations compared with the lowest-risk group (ED = 4).

The association between sweet categories and eating disorder risk was significant for the full sample (χ^2^[27] = 48.04, *p* = 0.008, Cramér’s V = 0.11, indicating a small effect). However, this result must be interpreted with substantial caution, given that (a) the distribution is extremely skewed toward the lowest-risk category (84.2%), (b) several cells in Table 4 contain counts below 5 (e.g., ED = 1 across multiple categories), which violates chi-square assumptions, and (c) within-nation analyses were significant only for the English-speaking sample. These findings should therefore be considered preliminary and exploratory. Participants with higher eating disorder risk (ED = 1 or 2) were overrepresented in the Negative/Health category: 25.0% of ED = 1 and 20.0% of ED = 2 responses fell in this category, compared with only 7.9% of ED = 4 responses. Craving/Desire was also more prevalent among ED = 1 participants (10.0%) than among ED = 4 (2.1%) (Figure 3). Within-nation analyses were significant only for the English-speaking sample (χ^2^[27] = 42.05, *p* = 0.033), whereas the Hungarian (*p* = 0.485) and Spanish (*p* = 0.677) subsamples did not reach significance, partly due to small cell sizes in the higher-risk groups.

The full-sample association between sweets categories and BMI category was also significant (χ^2^[27] = 42.71, *p* = 0.028, Cramér’s V = 0.11), but within-nation analyses were not significant (English *p* = 0.347; Hungarian *p* = 0.403; Spanish *p* = 0.847), indicating that the overall effect was likely driven by between-nation differences in both BMI distributions and association patterns rather than reflecting a genuine within-culture link between sweet-food cognitions and body weight. The association between sweets categories and household size was not significant in the full sample (χ^2^[27] = 33.11, *p* = 0.194) or in any within-nation analysis. Full within-nation tables for BMI, eating disorder risk, and household size are provided in the Supplementary Materials.

## 4. Discussion

### 4.1. Cultural Embedding of Sweet Food Associations

The present study revealed substantial differences across language groups in the most accessible lexical associations evoked by “sweets” across Hungarian, English, and Spanish communities. Using a refined 10-category coding scheme, the dominant pattern was clear: Chocolate dominated Hungarian responses (31.5%), while English speakers distributed their associations more broadly across Baked goods/Desserts, Sugar/Candy, and Mixed food items. Spanish speakers showed relatively higher proportions of Positive emotion and Food (general) responses. This is consistent with broader evidence that food-related mental representations are deeply embedded in cultural and experiential context [13,18,23,24].

However, it is important to acknowledge that cross-language differences in FWA responses may partly reflect lexical accessibility, word frequency, and translation asymmetries rather than deeper cognitive structures. The stimulus term is not semantically equivalent across the three languages: Hungarian “édesség” is a broad confectionery category term that may preferentially activate specific confectionery products; English “sweets” may carry evaluative connotations; and Spanish “dulce” exhibits polysemy extending to interpersonal warmth. These linguistic differences represent a fundamental interpretive challenge that limits the strength of cross-cultural conclusions.

The dominance of chocolate in Hungarian responses likely reflects both the high cultural salience of chocolate confectionery in Hungary and a phonological accessibility effect: “csoki”, the colloquial Hungarian diminutive, is a compact two-syllable prime particularly susceptible to rapid automatic activation. As noted by Tiffany [4], automatic cognitive schemata are activated rapidly in response to cues; the phonological compactness of “csoki” may lower the threshold for this activation relative to longer or less-rehearsed alternatives. The relatively high proportion of Negative/Health associations among English speakers (12.1%, compared to 6.3% among Hungarians and 7.0% among Spanish speakers) is consistent with the high penetration of anti-sugar public health messaging in Anglophone media [3,25], although this interpretation is conjectural and cannot be confirmed by the present data.

### 4.2. Sex Differences

Sex-related differences in association profiles were significant in the overall sample (Cramér’s V = 0.18, small-to-medium effect). Women more frequently associated sweets with Baked goods/Desserts, Mixed food items, and Negative/Health, while men showed higher proportions of Sugar/Candy and Chocolate responses. This is broadly consistent with evidence that women report stronger cravings for sweet foods and assign greater hedonic and health significance to food experiences [14,15]. The finding that women were overrepresented in the Negative/Health category suggests greater awareness of or concern about the health implications of sweet food consumption among women; they align with the results of a representative Polish study showing that women report greater health and nutrition concerns than men and are less likely to follow dietary patterns high in refined foods, sweets, sweetened beverages, fast food, and alcohol [26].

These sex differences in food cognition may also reflect broader gender-related differences in emotional regulation. Saccaro et al. (2023) [27] demonstrated that emotional regulation underlies gender differences in pathological eating behavior styles, with women displaying higher frequencies of emotional eating compared to men. The greater representation of women in the Negative/Health and Craving/Desire categories in the present study may thus partly reflect gender-specific emotional processing of food cues, including heightened health vigilance and emotion-driven food appraisal, rather than simple differences in awareness. Future research should investigate whether emotional regulation mediates the relationship between sex and food-related cognitive associations.

### 4.3. BMI and Eating Disorder Risk

The association between sweets categories and eating disorder risk, while statistically significant in the full sample (Cramér’s V = 0.11), should be interpreted as an exploratory finding given the severe limitations of the single-item measure and extreme distributional skew. Participants at higher eating disorder risk were descriptively overrepresented in the Negative/Health category, which may suggest that individuals with self-reported eating concerns tend to frame sweets in terms of health concern, dietary restriction, or transgression rather than in terms of specific food products or sensory pleasure. Consistent with our findings, a study with Chinese university students [28] suggests that those with greater eating-related vulnerability tend to approach food through a health- and restriction-oriented lens. This pattern is directionally consistent with cognitive-behavioral models of eating disorders, which emphasize the role of maladaptive food cognitions in maintaining dietary restriction and binge-purge cycling [29]. However, given the non-validated measure, extreme skew, and failure to replicate within-nation, these findings cannot support clinical claims and require replication with validated instruments (e.g., EAT-26, SCOFF) before any conclusions about vulnerability markers can be drawn.

BMI distributions differed markedly across nations, with the English-speaking sample showing substantially higher obesity prevalence (30.3%) than the Hungarian (8.9%) or Spanish (6.8%) samples. These findings are consistent with a pan-European projection study [30]. The full-sample association between sweet categories and BMI was statistically significant but had a small effect size (Cramér’s V = 0.11), and within-nation analyses were uniformly non-significant. This pattern strongly suggests that the overall effect is an artifact of between-nation differences in both BMI distributions and language-specific patterns of association, rather than reflecting a genuine within-culture link between sweet-food cognitions and body weight.

The null finding for household size further suggests that the meaning attributed to sweets is shaped primarily by cultural and individual psychological factors rather than by household context. However, at least one study has shown a positive association between household size and sweet consumption [31]. Guided by the Social Ecological Model, future research could examine whether the consumption and associated meanings of sweets differ between smaller and larger households [32,33].

### 4.4. Limitations

Several important limitations constrain the interpretability of the present findings, and their cumulative impact should be carefully considered. Firstly, the convenience sampling strategy via social media produces a self-selected, non-representative sample with unknown demographic biases. The heavy female skew (68% overall, 84% in the English sample) limits generalizability and the feasibility of sex-stratified analyses. Secondly, the language group was used as a proxy for cultural community, which conflates national, ethnic, and linguistic identities. Thirdly, the stimulus term “sweets” is not semantically equivalent across the three languages, and observed cross-cultural differences may partly reflect lexical accessibility and translation asymmetries rather than deeper cognitive structures. Fourthly, single-item FWA captures only the most salient association and does not provide information about the broader structure of food-related semantic networks. The absence of a time constraint and the allowance of multi-word responses reduce standardization. Fifthly, BMI was calculated from self-reported height and weight, which introduces measurement error and known systematic bias (height overestimation, weight underestimation). Sixthly, eating disorder risk was assessed with a single non-validated screening item that conflates diagnosis, treatment status, and subjective concern. The extreme skewness (84.2% in the lowest-risk category) and the resulting small cell counts compromise the chi-square assumptions and preclude reliable multivariate modeling. This is a central limitation that prevents clinical conclusions. Seventhly, the statistical analysis relies exclusively on bivariate chi-square tests without correction for multiple testing or multivariate modeling to control for confounders (e.g., the simultaneous effects of language, sex, and BMI). The absence of such controls means that observed associations may reflect confounded relationships. Collectively, these are not minor methodological caveats but central limitations that constrain the validity and generalizability of the findings.

## 5. Conclusions

This exploratory, cross-sectional study demonstrates that the most accessible cognitive associations with “sweets” vary significantly across Hungarian, English, and Spanish-speaking communities, with medium effect sizes (Cramér’s V = 0.28). Chocolate was the dominant association in Hungarian responses, while English speakers showed more distributed patterns across desserts, sugar/candy, and health-related concerns. Sex differences were significant but small to medium in magnitude (V = 0.18), with women more frequently producing health-related and dessert-specific associations. An exploratory association between sweets categories and self-reported eating disorder risk was observed (V = 0.11), with Negative/Health responses overrepresented among higher-risk participants; however, this finding is based on a non-validated single-item measure with extreme distributional skew and requires replication with validated instruments before any clinical implications can be considered. The cross-cultural findings underscore the importance of culturally sensitive approaches to dietary research and the need for future studies that employ representative samples, validated measures, multivariate designs, and careful attention to cross-linguistic equivalence of stimulus terms.

## Figures and Tables

**Table 1:** Frequency of Meaning Categories by Language Group (*N* = 1349).

Category	English	Hungarian	Spanish	Total
Baked goods/Desserts	105	58	80	243
Chocolate	40	145	42	227
Craving/Desire	16	12	5	33
Food (general)	8	8	26	42
Mixed food items	101	38	17	156
Negative/Health	66	29	24	119
Other	7	10	7	24
Positive emotion	85	100	67	252
Reward/Comfort	11	8	3	22
Sugar/Candy	105	53	73	231
Total	544	461	344	1349

Note. χ^2^(18) = 210.05, *p* < 0.001, Cramér’s V = 0.28.

**Table 2:** Frequency of Meaning Categories by Language Group and Sex.

	EN W	EN M	HU W	HU M	ES W	ES M
Baked goods/Desserts	91	14	44	14	41	39
Chocolate	31	8	90	54	23	19
Craving/Desire	12	4	7	5	4	1
Food (general)	8	0	5	3	7	19
Mixed food items	87	14	27	11	7	10
Negative/Health	59	6	21	8	12	12
Other	5	2	8	2	3	4
Positive emotion	76	9	66	34	41	26
Reward/Comfort	7	4	7	1	1	2
Sugar/Candy	82	22	21	32	24	49
Total	458	83	296	164	163	181

Note. EN = English; HU = Hungarian; ES = Spanish; W = Women; M = Men. Overall χ^2^(9) = 43.72, *p* < 0.001, Cramer’s V = 0.18 (sex main effect, excluding ‘Other sex,’ *n* = 4).

**Table 3:** BMI Category Distribution by Language Group (*n* = 1199).

BMI Category	English	Hungarian	Spanish	Total
Underweight	7	33	9	49
Healthy	163	315	243	721
Overweight	109	70	65	244
Obese	121	41	23	185
Total	400	459	340	1199

Note. χ^2^(6) = 157.17, *p* < 0.001, Cramer’s V = 0.26. UW = Underweight (BMI < 18.5); OW = Overweight (BMI 25.0–29.9); Obese = BMI ≥ 30.0. *n* = 150 participants were excluded due to missing data.

**Table 4:** Sweets Category × Eating Disorder Risk—Full Sample (*N* = 1349).

Sweets Category	ED = 1	ED = 2	ED = 3	ED = 4	Total
Baked goods/Desserts	2	8	27	206	243
Chocolate	2	3	27	195	227
Craving/Desire	2	2	5	24	33
Food (general)	0	1	1	40	42
Mixed food items	2	7	10	137	156
Negative/Health	5	13	11	90	119
Other	0	3	2	19	24
Positive emotion	5	11	26	210	252
Reward/Comfort	0	1	3	18	22
Sugar/Candy	2	16	16	197	231
Total	20	65	128	1136	1349

Note. ED = eating disorder risk (1 = highest, 4 = lowest). χ^2^(27) = 48.04, *p* = 0.008, Cramer’s V = 0.11. The distribution is heavily skewed toward ED = 4 (*n* = 1136, 84.2%).

## Data Availability

The data are available at the Mendeley data repository (DOI: 10.17632/hfyg854jj2.1).

## Supplementary Materials

The following supporting information can be downloaded at https://www.mdpi.com/article/10.3390/nu18111771/s1, Table S1: BMI Category Distribution by Sex; Table S2: Sweets Category × BMI Category—Full Sample; Tables S3–S5: Sweets Category × BMI Category by nation; Tables S6–S8: Sweets Category × Eating Disorder Risk by nation; Tables S9–S12: Sweets Category × Household Size by nation.
