# Supplementary material for: Cultural and Sex-Related Differences in Free-Word Associations with “Sweets”: A Multinational Online Study
Source: Nutrients. 2026 May 30;18(11):1771. doi: 10.3390/nu18111771 (PMC13258868; doi:10.3390/nu18111771)
Supplement: Supplementary file 1 [file nutrients-18-01771-s001.zip › nutrients-4268604-supplementary.pdf]

SUPPLEMENTARY MATERIAL

Table S1. BMI Category Distribution by Sex (n = 1,195).

BMI Category	Female	Male	Total
Underweight	41	8	**49**
Healthy	456	264	**720**
Overweight	139	105	**244**
Obese	149	33	**182**
**Total**	**785**	**410**	**1195**

Note. $\chi^2(3) = 38.18$, $p < .001$. Four participants of the other sex were excluded.

Table S2. Sweets Category × BMI Category—Full Sample with Complete Data.

Sweets Category	UW	Healthy	OW	Obese	Total
Baked goods/Desserts	7	128	45	33	**213**
Chocolate	12	134	43	27	**216**
Craving/Desire	0	13	11	5	**29**
Food (general)	3	32	4	1	**40**
Mixed food items	2	80	25	23	**130**
Negative/Health	2	59	18	22	**101**
Other	2	10	9	1	**22**
Positive emotion	13	128	50	41	**232**
Reward/Comfort	2	10	2	3	**17**
Sugar/Candy	6	127	37	29	**199**
**Total**	**49**	**721**	**244**	**185**	**1199**

Note. $\chi^2(27) = 42.71$, $p = .028$. UW = Underweight; OW = Overweight.

Table S3. Sweets Category × BMI Category—English (n = 400).

Sweets Category	UW	Healthy	OW	Obese	Total
Baked goods/Desserts	2	31	19	24	**76**
Chocolate	0	13	11	7	**31**
Craving/Desire	0	4	5	4	**13**
Food (general)	0	4	2	0	**6**
Mixed food items	0	36	18	21	**75**
Negative/Health	2	20	8	18	**48**
Other	0	0	4	1	**5**
Positive emotion	1	22	20	22	**65**
Reward/Comfort	1	3	1	2	**7**
Sugar/Candy	1	30	21	22	**74**
**Total**	**7**	**163**	**109**	**121**	**400**

Note. $\chi^2(27) = 29.30$, $p = .347$, ns.

Table S4. Sweets Category × BMI Category—Hungarian (n = 459).

Sweets Category	UW	Healthy	OW	Obese	Total

Baked goods/Desserts	3	45	8	2	**58**
Chocolate	11	93	24	17	**145**
Craving/Desire	0	6	4	1	**11**
Food (general)	2	5	1	0	**8**
Mixed food items	2	30	4	2	**38**
Negative/Health	0	21	5	3	**29**
Other	2	5	3	0	**10**
Positive emotion	8	65	14	13	**100**
Reward/Comfort	1	5	1	1	**8**
Sugar/Candy	4	40	6	2	**52**
**Total**	**33**	**315**	**70**	**41**	**459**

Note. $\chi^2(27) = 28.15$, $p = .403$, ns.

Table S5. Sweets Category × BMI Category—Spanish ($n = 340$).

Sweets Category	UW	Healthy	OW	Obese	Total
Baked goods/Desserts	2	52	18	7	**79**
Chocolate	1	28	8	3	**40**
Craving/Desire	0	3	2	0	**5**
Food (general)	1	23	1	1	**26**
Mixed food items	0	14	3	0	**17**
Negative/Health	0	18	5	1	**24**
Other	0	5	2	0	**7**
Positive emotion	4	41	16	6	**67**
Reward/Comfort	0	2	0	0	**2**
Sugar/Candy	1	57	10	5	**73**
**Total**	**9**	**243**	**65**	**23**	**340**

Note. $\chi^2(27) = 19.60$, $p = .847$, ns.

Table S6. Sweets Category × Eating Disorder Risk—English ($n = 544$).

Sweets Category	ED=1	ED=2	ED=3	ED=4	Total
Baked goods/Desserts	1	5	15	84	**105**
Chocolate	0	1	9	30	**40**
Craving/Desire	2	1	2	11	**16**
Food (general)	0	1	1	6	**8**
Mixed food items	2	6	7	86	**101**
Negative/Health	5	10	9	42	**66**
Other	0	1	1	5	**7**
Positive emotion	1	7	13	64	**85**
Reward/Comfort	0	0	1	10	**11**
Sugar/Candy	1	13	9	82	**105**
**Total**	**12**	**45**	**67**	**420**	**544**

Note. $\chi^2(27) = 42.05$, $p = .033$.

Table S7. Sweets Category × Eating Disorder Risk—Hungarian (n = 461).

Sweets Category	ED=1	ED=2	ED=3	ED=4	Total
Baked goods/Desserts	0	2	7	49	**58**
Chocolate	1	1	15	128	**145**
Craving/Desire	0	1	2	9	**12**
Food (general)	0	0	0	8	**8**
Mixed food items	0	1	3	34	**38**
Negative/Health	0	3	1	25	**29**
Other	0	2	0	8	**10**
Positive emotion	2	3	12	83	**100**
Reward/Comfort	0	1	1	6	**8**
Sugar/Candy	1	3	5	44	**53**
**Total**	**4**	**17**	**46**	**394**	**461**

Note. $\chi^2(27) = 26.61$, $p = .485$, ns.

Table S8. Sweets Category × Eating Disorder Risk—Spanish (n = 344).

Sweets Category	ED=1	ED=2	ED=3	ED=4	Total
Baked goods/Desserts	1	1	5	73	**80**
Chocolate	1	1	3	37	**42**
Craving/Desire	0	0	1	4	**5**
Food (general)	0	0	0	26	**26**
Mixed food items	0	0	0	17	**17**
Negative/Health	0	0	1	23	**24**
Other	0	0	1	6	**7**
Positive emotion	2	1	1	63	**67**
Reward/Comfort	0	0	1	2	**3**
Sugar/Candy	0	0	2	71	**73**
**Total**	**4**	**3**	**15**	**322**	**344**

Note. $\chi^2(27) = 23.14$, $p = .677$, ns.

Table S9. Sweets Category × Household Size—Full Sample (N = 1,349).

Sweets Category	PH=1	PH=2	PH=3	PH=4	Total
Baked goods/Desserts	29	90	109	15	**243**
Chocolate	26	56	115	30	**227**
Craving/Desire	5	9	14	5	**33**
Food (general)	6	10	18	8	**42**
Mixed food items	19	61	59	17	**156**
Negative/Health	16	46	46	11	**119**
Other	0	6	11	4	**24**
Positive emotion	44	71	108	29	**252**
Reward/Comfort	4	7	8	3	**22**
Sugar/Candy	28	73	105	25	**231**
**Total**	**180**	**429**	**593**	**147**	**1349**

Note. $\chi^2(27) = 33.11$, $p = .194$, ns. PH = People in Household.

Table S10. Sweets Category × Household Size—English (n = 544).

Sweets Category	PH=1	PH=2	PH=3	PH=4	Total
Baked goods/Desserts	10	54	38	3	**105**
Chocolate	4	16	14	6	**40**
Craving/Desire	4	5	6	1	**16**
Food (general)	1	5	1	1	**8**
Mixed food items	15	47	28	11	**101**
Negative/Health	6	28	24	8	**66**
Other	2	2	2	1	**7**
Positive emotion	18	41	24	2	**85**
Reward/Comfort	3	6	2	0	**11**
Sugar/Candy	18	48	34	5	**105**
**Total**	**81**	**252**	**173**	**38**	**544**

Note. $\chi^2(27) = 32.82$, $p = .203$, ns.

Table S11. Sweets Category × Household Size—Hungarian (n = 461).

Sweets Category	PH=1	PH=2	PH=3	PH=4	Total
Baked goods/Desserts	8	11	33	6	**58**
Chocolate	18	32	80	15	**145**
Craving/Desire	0	3	5	4	**12**
Food (general)	0	1	5	2	**8**
Mixed food items	2	13	19	4	**38**
Negative/Health	5	12	10	2	**29**
Other	0	3	4	3	**10**
Positive emotion	15	18	49	18	**100**
Reward/Comfort	0	1	4	3	**8**
Sugar/Candy	5	9	32	7	**53**
**Total**	**53**	**103**	**241**	**64**	**461**

Note. $\chi^2(27) = 35.35$, $p = .130$, ns.

Table S12. Sweets Category × Household Size—Spanish (n = 344).

Sweets Category	PH=1	PH=2	PH=3	PH=4	Total
Baked goods/Desserts	11	25	38	6	**80**
Chocolate	4	8	21	9	**42**
Craving/Desire	1	1	3	0	**5**
Food (general)	5	4	12	5	**26**
Mixed food items	2	1	12	2	**17**
Negative/Health	5	6	12	1	**24**
Other	1	1	5	0	**7**
Positive emotion	11	12	35	9	**67**
Reward/Comfort	1	0	2	0	**3**
Sugar/Candy	5	16	39	13	**73**
**Total**	**46**	**74**	**179**	**45**	**344**

Note. $\chi^2(27) = 25.39$, $p = .553$, ns.
